# Bichromatic tetraphasic full-field optical coherence microscopy

**DOI:** 10.1117/1.JBO.29.S2.S22704

**Published:** 2024-03-25

**Authors:** Rishyashring R. Iyer, Mantas Žurauskas, Yug Rao, Eric J. Chaney, Stephen A. Boppart

**Affiliations:** a University of Illinois Urbana-Champaign, Beckman Institute for Advanced Science and Technology, Urbana, Illinois, United States; b University of Illinois Urbana-Champaign, Department of Electrical and Computer Engineering, Urbana, Illinois, United States; c University of Illinois Urbana-Champaign, Department of Bioengineering, Urbana, Illinois, United States; d University of Illinois Urbana Champaign, NIH/NIBIB P41 Center for Label-free Imaging and Multiscale Biophotonics (CLIMB), Urbana, Illinois, United States; e University of Illinois Urbana-Champaign, Cancer Center at Illinois, Urbana, Illinois, United States; f University of Illinois Urbana-Champaign, Carle Illinois College of Medicine, Urbana, Illinois, United States

**Keywords:** phase imaging, spectroscopic imaging, optical coherence microscopy, biological dynamics

## Abstract

**Significance:**

Full-field optical coherence microscopy (FF-OCM) is a prevalent technique for backscattering and phase imaging with epi-detection. Traditional methods have two limitations: suboptimal utilization of functional information about the sample and complicated optical design with several moving parts for phase contrast.

**Aim:**

We report an OCM setup capable of generating dynamic intensity, phase, and pseudo-spectroscopic contrast with single-shot full-field video-rate imaging called bichromatic tetraphasic (BiTe) full-field OCM with no moving parts.

**Approach:**

BiTe OCM resourcefully uses the phase-shifting properties of anti-reflection (AR) coatings outside the rated bandwidths to create four unique phase shifts, which are detected with two emission filters for spectroscopic contrast.

**Results:**

BiTe OCM overcomes the disadvantages of previous FF-OCM setup techniques by capturing both the intensity and phase profiles without any artifacts or speckle noise for imaging scattering samples in three-dimensional (3D). BiTe OCM also utilizes the raw data effectively to generate three complementary contrasts: intensity, phase, and color. We demonstrate BiTe OCM to observe cellular dynamics, image live, and moving micro-animals in 3D, capture the spectroscopic hemodynamics of scattering tissues along with dynamic intensity and phase profiles, and image the microstructure of fall foliage with two different colors.

**Conclusions:**

BiTe OCM can maximize the information efficiency of FF-OCM while maintaining overall simplicity in design for quantitative, dynamic, and spectroscopic characterization of biological samples.

## 1 Introduction

Since its advent in the 1990s,^1^ optical coherence tomography (OCT) has become ubiquitous in biomedical imaging, ranging from ophthalmology to endoscopy.^2^ There is but one universal principle for OCT: optical amplification of backscattering and axial sectioning through coherence gating via interferometric detection of spectrally broad beams. The apparent simplicity of this principle has enabled dozens of OCT techniques such as spectral-domain OCT,^3^ time-domain OCT,^1^ *en face* OCT,^4^ swept-source OCT,^5^ spectroscopic OCT,^6^ optical coherence elastography,^7^ and interferometric synthetic aperture microscopy.^8^ Most imaging scientists describe OCT in one of two ways: as an alternative to confocal microscopy that avoids the pinhole or as an optical analog to ultrasound imaging. Ultimately, the goal of an OCT setup is to estimate the backscattered signal localized in three-dimensional (3D) space, namely, x, y, and z (direction of light propagation). While localization in z is achieved inherently from coherence gating, there are several ways to achieve localization in x and in y, such as raster scanning of a focusing beam, line focus,^9^ or full-field detection^10^ with wide-field illumination. Early implementations of OCT typically relied on raster scanning like confocal microscopy. However, by the early 2000s, the stability and speed offered by full-field OCT led to the realization of several full-field OCT setups.^10^^–^^15^

Full-field OCT is typically used to image a single plane in a 3D sample (with some notable exceptions^13^). For high NA applications of optical coherence microscopy (OCM), full-field OCM (FF-OCM) has better data efficiency than Fourier-domain OCM setups, where the axial range of acquisition surpasses the Rayleigh range of high-NA beams by an order of magnitude. Theoretically, for the complete reconstruction of FF-OCM images, the interference must be detected at two to four different phase shifts, preferably separated by π/2 radians. Previous studies have solved this in four ways. In the first and the most widely used method, the reference mirror is physically or optically displaced (using a piezoelectric^16^ or modulating element,^14^ respectively) to create phase differences between the sample and reference beams, which requires multiple captures of the interferogram to generate one OCM image. In an alternative second method, the reference beam is spatially modulated to achieve off-axis OCM to get a complete reconstruction of OCM images by demodulation in the spatial-frequency domain with a single-shot acquisition.^17^^,^^18^ However, this requires a spatially coherent beam that induces unwanted speckle artifacts in the resultant images. The third method questions the necessity of complete reconstruction and instead uses the inherent fluctuations in the scattering of biological samples to generate a dynamic contrast (called dynamic OCM^19^^,^^20^). While the first two methods yield complex-valued images that can be used for further quantitative analysis, dynamic OCM only generates real-valued intensity fluctuations, thereby limiting applications to live biological samples. Dynamic contrast in OCT was implemented beyond full-field OCM even on spectral-domain OCT for imaging airway^21^ and embryo microstructures,^22^^,^^23^ where different components within the sample were apparent at different frequency components. Finally, we have shown that by using a λ/8 waveplate one can create four simultaneous phase differences in a randomly polarized beam that can later be spatially separated with a polarizing beam splitter (PBS) and detected with a single sensor.^24^ While this technique avoids multiple captures and speckle noise and provides a complete reconstruction of the backscattered optical field, the limited utility of λ/8 waveplates for spectrally broad light sources and the wavefront error introduced by the Soleil-Babinet compensator used as a λ/8 waveplate presents a challenge in imaging 3D scattering samples. For spatially incoherent sources, such as an LED, the wavefront errors caused by the variable waveplate exacerbated the wavefront errors from the scattering samples that limited the degree of interference spatially.^13^

The popularity of OCT in biomedical imaging could also be attributed to its ability to extract quantitative functional information from biological samples. Since OCT/OCM generates the complex-valued backscattered field rather than just the real-valued intensity profiles, the underlying dynamics can be quantified as a function of the optical phase, which can later be related to nanoscale displacements,^7^ ion flux,^25^ or blood flow.^26^^,^^27^ Functional OCT maximizes the information efficiency of the technique by providing biologically relevant properties beyond just structural content. Among that, spectroscopic OCT has utilized the space-spectrum Fourier relationship to estimate the spectral absorption and backscattering profiles.^6^ Practically, spectroscopic OCT has been used to observe blood oxygenation levels by looking at the differences in the absorption of oxygenated and deoxygenated hemoglobin.^28^^,^^29^

In this paper, we present a simple setup for pseudo-spectroscopic FF-OCM by detecting the four unique phase shifts created by the spectrally mismatched anti-reflection (AR) coatings of a beam splitter placed at a subtle tilt under two different spectral windows. First, measuring the optical field at two spectral windows is sufficient to realize the practical utilization of FF-OCM, especially for estimating blood oxygenation levels. Second, two-phase shifts are enough to reconstruct the optical phase in FF-OCM. Therefore, our setup, called bichromatic tetraphasic OCM (BiTe OCM), can maximize the information efficiency of FF-OCM while maintaining overall simplicity in design. BiTe OCM generates three complementary contrasts: optical intensity changes from biological dynamics, reconstructed optical phase for each color, and pseudo-spectroscopic differences. We have demonstrated our setup by observing the cellular dynamics of *in vitro* samples, the hemodynamics of *ex vivo* breast tumors, and the dynamic spectroscopic properties of fall foliage.

## 2 Design and Development of BiTe OCM

### 2.1 Principle

BiTe OCM utilizes the phase-shifting properties of AR coatings on optical surfaces. AR coatings are typically thin films of materials with refractive indices lower than the optical element but higher than air (∼1.3 to 1.4), with a thickness typically one-quarter of the optical wavelength in air.^30^ The reflected beams at the two interfaces of the coatings are shifted by half a wavelength and interfere destructively in the far field. Multiple such surfaces of varying refractive indices are stacked to increase the operational bandwidth of the AR coatings.

When used away from their rated wavelengths, i.e., at the “wrong” or spectrally mismatched AR coating, the reflected beam does not interfere destructively but is rather shifted by an arbitrary phase with respect to the transmitted beam. For instance, when a visible beam centered at 535 nm enters an optical element with an AR coating rated for NIR wavelengths (650 to 1050 nm), not only is the reflectance at the surface >10% but there is an apparent phase shift to the transmitted beam with respected to the reflected beam. While the exact specifications of these coatings may vary from one manufacturer to another, the principle remains the same.

**Fig. 1:**
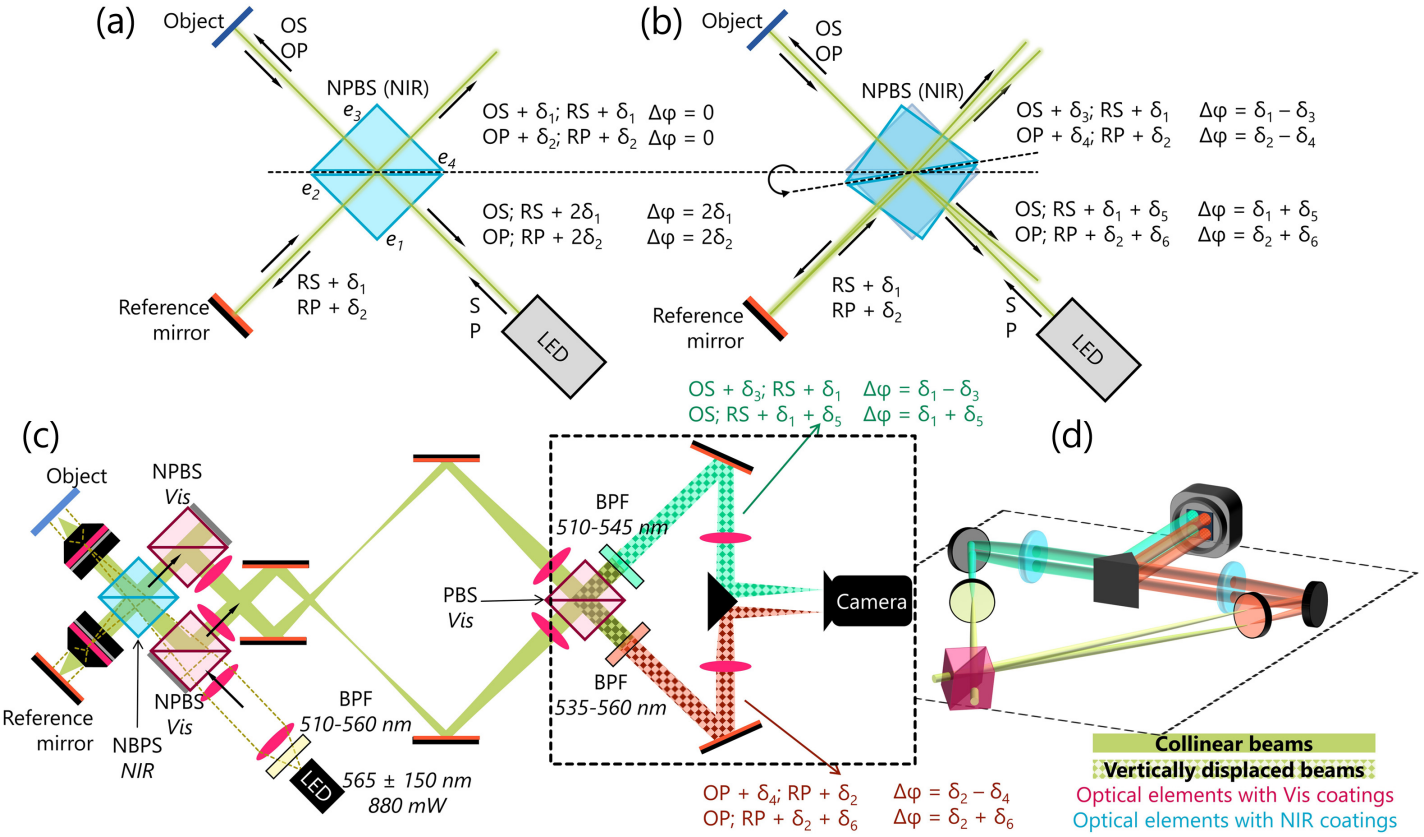
Principle and system setup of BiTe OCM. (a) and (b) Phase shifts from the sample (Sam) and reference (Ref) arms for S- and P-polarized components of a beam (visible) when passing through a beam-splitter cube of the spectrally mismatched (NIR) coating a- incident normally, and b- incident at an angle. (c) System setup of BiTe OCM, where the interference between the sample and the reference is captured at four unique phase shifts by passing it through a beam-splitter cube of the spectrally mismatched (NIR) coating. (d) 3D view of the highlighted region in c, showing the vertical displacements of the beams to separately detect each phase shift combination simultaneously. NPBS, non-polarizing beam-splitter; BPF, bandpass filter; PBS, polarizing beam-splitter; Vis, visible light (400 to 700 nm).

Consider when an optical beam passes twice through a beam splitter with the spectrally mismatched AR coating, as expected in a Michelson interferometer [Fig. 1(a)]. For an S-polarized beam entering through surface $e_{1}$ of the beam splitter, there are two interference patterns emerging out of the surfaces $e_{1}$ and $e_{4}$. The beam emerging out of $e_{4}$ is the interference pattern between the sample beam reflected once by the beam splitter and the reference beam reflected once by the beam splitter, each shifted by an arbitrary phase, $\delta_{1}$. The overall phase difference between the sample and reference beams at $e_{4}$ is, therefore, zero. The beam emerging out of $e_{1}$ is the interference pattern between the object beam never reflected by the beam splitter and the reference beam reflected twice by the beam splitter and shifted by a phase of $2\delta_{1}$. The overall phase difference between the object and reference beams at $e_{1}$ is, therefore, $2\delta_{1}$. For a P-polarized beam, the phase differences at $e_{4}$ and $e_{1}$ are 0 and $2\delta_{2}$, respectively. The case presented in Fig. 1(a) is for an ideal scenario in which all beams are incident at normal angles, where the interference patterns have three unique phase differences: 0, 0, $2\delta_{1}$, and $2\delta_{2}$. If the beam splitter was rotated slightly, the interference patterns could have four unique phase differences due to the differences in their angles of incidence on the beam splitter: $\delta_{1}-\delta_{3}$, $\delta_{2}-\delta_{4}$, $\delta_{1}+\delta_{5}$, and $\delta_{2}+\delta_{6}$ [Fig. 1(b)]. Since the two interference patterns emerging out of each beam splitter phase are of different polarizations, they can be separated spatially with a PBS further down the beam path and spectrally separated with two bandpass filters. Finally, each beam can be recombined to be detected simultaneously with a single detector.

### 2.2 System Setup

Figure 1(c) describes the setup of BiTe OCM based on these principles, which are adapted from a previous setup.^24^ BiTe OCM is sourced by a 565-nm LED (M565L3, Thorlabs Inc., NJ) whose bandwidth is truncated by a 510 to 560-nm bandpass filter (Semrock, NY). A non-polarizing beam splitter (NPBS) cube rated for NIR light (47% reflectance and transmission between 650 and 1050 nm, with 17% to 30% reflectance at 535 nm) is used as the primary beam splitter in the interferometer (CCM1-BS014, Thorlabs Inc.). The four interference patterns are spatially separated from the incident beams and from each other using two NPBS (BS013, Thorlabs Inc., rated for 420 to 680 nm with 47% to 52% reflectance at 535 nm) and one PBS (CCM1-PBS251, Thorlabs Inc., rated for 420 to 680 nm) of the “correct” AR coating [Fig. 1(d)] and simultaneously detected with a single camera (Q-2HFW-Hm/CXP-6-0.4, Adimec, Eindhoven, Netherlands) interfaced to the computer via CoaXPress to a PCIe-based frame grabber (CoaxLink Quad G3, Euresys SA, Seraing, Belgium). Data acquisition is performed by a custom LabVIEW (National Instruments Corp, TX) software using the CoaxLink API. The objective lens has an NA of 0.8 (LUMPLFLN40XW, Olympus Life Sciences, Tokyo, Japan), yielding a transverse resolution of 0.5  μm. The axial resolution for each detection bandwidth is ∼5  μm. The images in this study were captured at 15 to 20 frames per second, limited by the reflectivity of the samples at the maximum fluence of the LED.

### 2.3 Image Processing

**Fig. 2:**
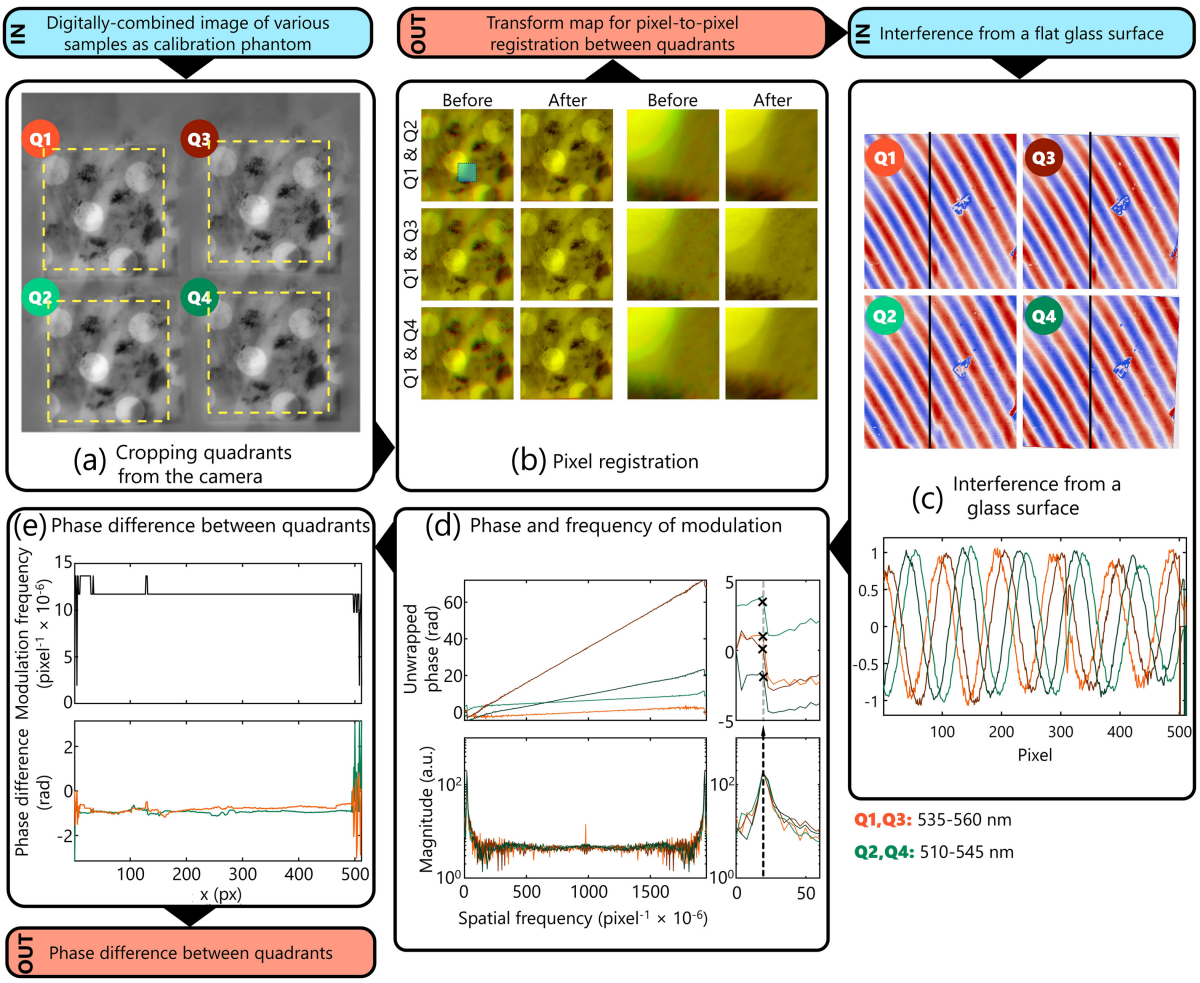
Calibration for reconstructing the phase in BiTE OCM. (a) First, the four quadrants of the images are cropped to obtain $I_{Q_{1}-Q_{4}}(x,y)$. (b) From an amalgamation of images from various samples, a pixel-pixel registration map is created to ensure maximum overlap; validated by observing the overlap before and after pixel-registration correction between quadrants two and four registered to quadrant 1. The area shaded in green in the first column is highlighted for each condition in the third and fourth columns. The pixel registration map is stored for unwarping the quadrant in each subsequent sample. (c) The interference from a glass surface after pixel registration correction, with the plot below showing the modulation profile along the black lines in the image. (d) The modulation of each line in the image is analyzed in the spatial frequency domain to extract the phase at the modulation frequency, which is estimated as the spatial frequency with maximum amplitude in the Fourier space. (e) The modulation and phase differences extracted between [$Q_{1}$ and $Q_{3}$ (orange); $Q_{2}$ and $Q_{4}$ (green)] for each line are stored. The input images to these steps are highlighted in blue and the outputs in orange.

Extracting the three contrasts from BiTe OCM requires a series of calibration steps described in Fig. 2. First, various samples with different features are imaged in the object arm (with the reference arm blocked) and combined linearly to generate a single frame. Each quadrant ($Q_{m}∋m=\{1,2,3,4\}$) is cropped to have approximately the same fields of view and an equal number of pixels [Fig. 2(a)]. $Q_{1}$ and $Q_{3}$ are filtered using the 535 to 560 nm bandpass filter; $Q_{2}$ and $Q_{4}$ are filtered using the 510 to 545 nm bandpass filter. The transformation map from $Q_{1}$ to $Q_{2-4}$ was derived using the imregcorr() function in MATLAB (Mathworks Inc., v2022b) [Fig. 2(b)]. This correlation-based registration algorithm performed poorly on fractal-like or uniformly patterned samples, typical of test and calibration targets. This necessitated computationally combining frames of different objects into a single image, which was easier than devising a physical phantom with all these characteristics. The transform map, along with the coordinates for cropping, were performed after every alignment check in our prototype setup and were saved for the next steps. Next, the interference from a glass surface is imaged. A spatial modulation pattern in each quadrant is apparent due to the deviation from normal incidence at the beam splitter. First, the interference from each quadrant is cropped and registered to $Q_{1}$ using the imwarp() function in MATLAB and the previously saved transform maps [Fig. 2(c)]. The sinusoidal modulation in each line is Fourier transformed, from which the modulation frequency and phase at the modulation frequency, $\delta_{Q_{x}}(y)$, are noted [Fig. 2(d)]. An alternate method to phase estimation was also utilized, based on estimating the lag of maximum correlation coefficient. An equivalent sinusoidal with no phase lag was generated at the modulation frequency of every line and cross-correlated with the interferogram of each line. The corresponding phase of the sinusoid at the lag that has the maximum correlation coefficient was estimated as the phase of the modulation frequency for the line. Both methods yielded equivalent results. Since this estimation only covers half the angular space (0-π), the sign was estimated based on the reconstructed phase of a glass surface. The phase difference between quadrants of the same color, $\theta_{Q_{2}-Q_{4}}(y)$ and $\theta_{Q_{1}-Q_{3}}(y)$, are derived from subtracting the $\delta_{Q_{x}}(y)$ of the respective quadrants [Fig. 2(e)]. Finally, the images from the reference mirror with the sample arm blocked are acquired as background for each quadrant. The cropped images from each quadrant, $I_{Q_{x}}(x,y)$, are unwrapped, $I_{Q_{x}}^{U}(x,y)$. Then, each frame in each quadrant is background subtracted and normalized

$$I_{Q_{x}}^{N}(x,y)=\frac{I_{Q_{x}}^{U}(x,y)-B_{Q_{x}}^{U}(x,y)}{\sqrt{B_{Q_{x}}^{U}(x,y)}},$$ (1)

where B is the background image.

For each frame, the phase and the intensity can be mathematically reconstructed from two quadrants. Assuming the two quadrants, $Q_{m}$ and $Q_{n}$, of the same color with a relative shift of $\theta_{Q_{n}-Q_{m}}(y)$, the captured interferograms can be defined as

$$I_{Q_{n}}^{U}(x,y)=E_{Q_{n}}^{U2}(x,y)+B_{Q_{n}}^{U}(x,y)+2E_{Q_{n}}^{U}(x,y)\sqrt{B_{Q_{n}}^{U}(x,y)}\;\cos[\phi_{Q_{n}}(x,y)+\theta_{Q_{n}-Q_{m}}(y)]\text{ and}\;I_{Q_{m}}^{U}(x,y)=E_{Q_{m}}^{U2}(x,y)+B_{Q_{m}}^{U}(x,y)+2E_{Q_{m}}^{U}(x,y)\sqrt{B_{Q_{m}}^{U}(x,y)}\;\cos[\phi_{Q_{m}}(x,y)+0],$$ (2)

where E and ϕ are the backscattered sample signal intensity and phase, respectively. Assuming $E_{Q_{n}}^{U}(x,y)=E_{Q_{m}}^{U}(x,y)=E(x,y)$, $\phi_{Q_{n}}(x,y)=\phi_{Q_{m}}(x,y)=\phi (x,y)$, and $E^{2}(x,y)\to 0$, the backscattered signal intensity and phase can be defined as

$$\phi (x,y)=\tan^{-1}(\frac{I_{Q_{m}}^{N}(x,y)\cos[\theta_{Q_{n}-Q_{m}}(y)]-I_{Q_{n}}^{N}(x,y)}{I_{Q_{m}}^{N}(x,y)\sin[\theta_{Q_{n}-Q_{m}}(y)]})\;\text{and}\;E(x,y)=\frac{I_{Q_{m}}^{N}(x,y)}{\cos[\phi (x,y)]}=\frac{I_{Q_{n}}^{N}(x,y)}{\cos[\phi (x,y)+\theta_{Q_{n}-Q_{m}}(y)]}\;=\frac{1}{2}(\frac{I_{Q_{m}}^{N}(x,y)}{\cos[\phi (x,y)]}+\frac{I_{Q_{n}}^{N}(x,y)}{\cos[\phi (x,y)+\theta_{Q_{n}-Q_{m}}(y)]}).$$ (3)

For the dynamic OCM contrast, the cropped images from each quadrant are background subtracted and reshaped as image stacks where the third axis is a time series of 500 to 2000 frames. Each stack is Fourier transformed along the time-axis, certain frequency bands are cropped, and the stack is inverse Fourier transformed. Each frame in the inverted stack is unwarned using the parameters from the previous calibration. The mean of the magnitude is displayed as the dynamic OCM contrast for each quadrant; the average dynamic contrast for each color represents the pseudo-spectroscopic dynamic OCM contrast presented in the following sections. Since the phase reconstructed from Eq. (3) is often noisy, we devised a pseudo phase retrieval algorithm that is more robust to acquisition noise and sample dynamics. Assuming $S_{Q_{x}}$ is the average of $I_{Q_{x}}^{N}(x,y)$ for all pixels across the time series, the reconstructed pseudo phase for each color is defined as

$$\phi_{510-545\;\mathrm{nm}}(x,y)=\cos^{-1}(\frac{2I_{Q_{2}}^{N}(x,y)I_{Q_{4}}^{N}(x,y)}{S_{Q_{2}}S_{Q_{4}}}-\cos[\theta_{Q_{2}-Q_{4}}(y)]),\;\phi_{535-560\;\mathrm{nm}}(x,y)=\cos^{-1}(\frac{2I_{Q_{1}}^{N}(x,y)I_{Q_{3}}^{N}(x,y)}{S_{Q_{1}}S_{Q_{3}}}-\cos[\theta_{Q_{1}-Q_{3}}(y)]).$$ (4)

The complete derivation and rationale for this equation are described in Note S1 in the Supplementary Material. The stacks of the reconstructed phase for each color are Fourier transformed along the time-axis, the necessary frequency bands are cropped, and the stack is inverse Fourier transformed. For the samples against a bright background, such as cells cultured on a flat surface, the frequency bands 0.5 to 3 Hz were chosen. For samples with more diffused scattering, to reduce the noise from higher frequency components, the band between 0.5 and 2.25 Hz was considered. The standard deviation of the magnitude of the phase is displayed as the reconstructed phase contrast for each color. Additionally, the maximum instance of phase activity, quantified as the instance at which the phase difference between adjacent frames in the stack was maximum, is used in the later sections. Finally, overlays of these various contrasts at different colors help elucidate the capabilities of the setup.

## 3 Using BiTe OCM for Biological Imaging

### 3.1 Reconstructing the Intensity and Quantitative Phase in BiTe OCM

**Fig. 3:**
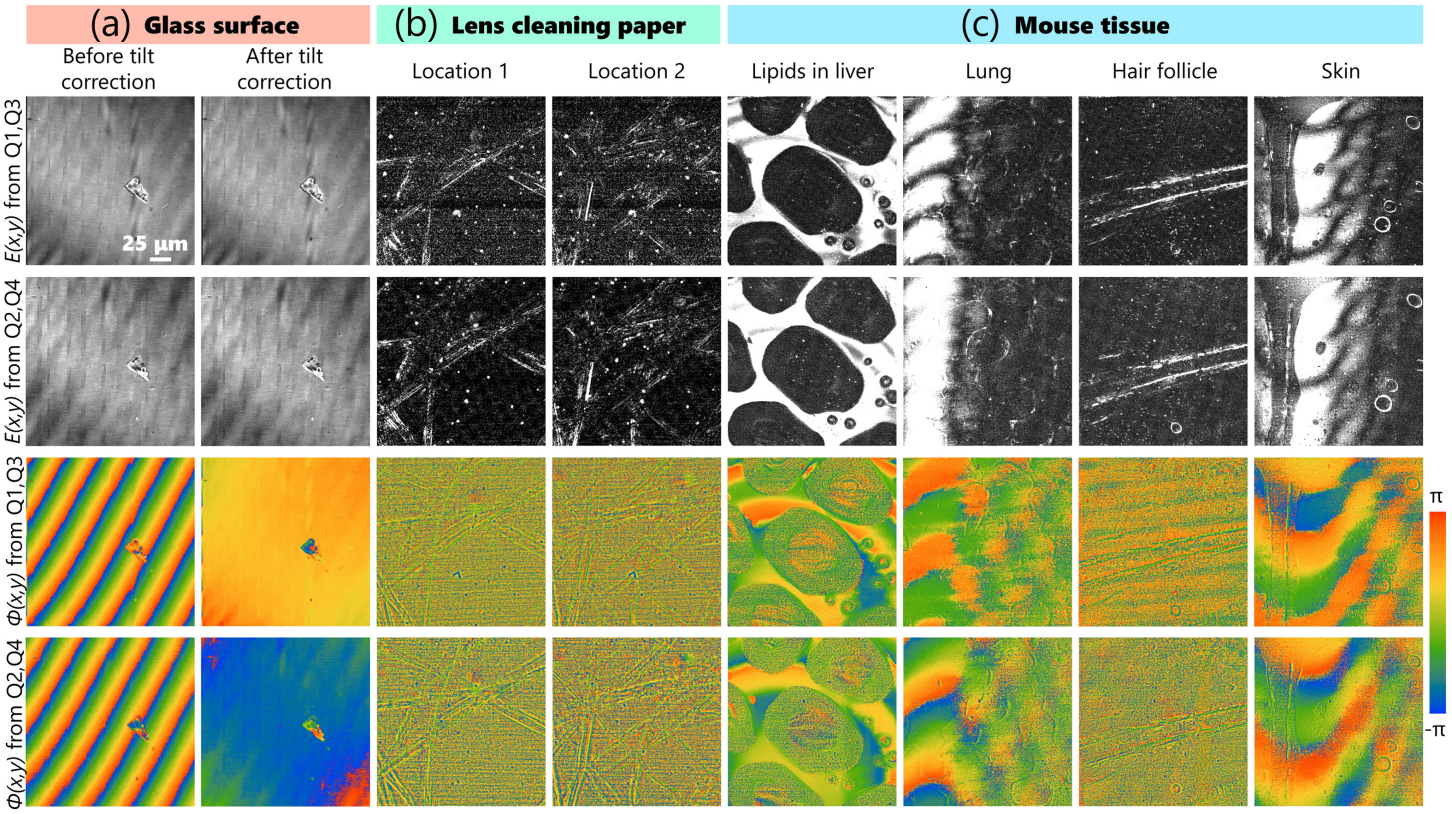
(a) Reconstructing the intensity and quantitative phase in BiTe OCM a glass surface, (b) lens cleaning papers, and (c) mouse tissues from each pair of quadrants. Scale bar: 25  μm.

Figure 3 highlights the quantitative phase and intensity reconstructed of each pair of quadrants imaging a flat glass surface, lens cleaning paper, and various organs from a mouse *ex vivo*. The mouse was euthanized by ${\mathrm{CO}}_{2}$ asphyxiation and the tissues were surgically resected and placed in an imaging dish with clear glass bottom containing ∼100  μL of freshly prepared phosphate-buffered saline, placed on ice, and imaged with a few hours of extraction. All animal procedures were conducted in accordance with protocols approved by the Illinois Institutional Animal Care and Use Committee at the University of Illinois at Urbana-Champaign and in compliance with the ARRIVE guidelines.

The phase of the glass surface can be reconstructed for every point in the image accurately due to nearly uniform scattering from the entire surface. While the tilt between the sample and reference signals is apparent in the first column of Fig. 3(a), it can be corrected by phase conjugation with a two-dimensional linear phase correction function, as seen in the second column. For sparsely scattering samples, such as the lens tissue and the hair follicles, only the regions with higher backscattered intensity have reliable phase values. This disparity in the retrieved phase values is also apparent in heterogeneous samples such as tissues, which could have both brightly and weakly scattering features within the axial section. Therefore, rather than using this “static” phase and intensity contrasts that are susceptible to noise, we instead use the dynamic intensity contrast and pseudo reconstructed phase in the rest of the paper, which yield better quality images.

### 3.2 Imaging Cellular Dynamics with BiTe OCM

Secondary cultures of NE-4C mouse neuroectodermal cells (CRL-2925, American Type Culture Collection, VA) were plated on a 35-mm glass-bottom Petri dish with a poly-D-lysine and grown in Eagle’s modified essential medium with a total of 4  μM L–glutamine (10009CV, Corning, NY), supplemented with 10% v/v fetal bovine serum (16140071, Thermo Fisher Scientific, MA) and 1% v/v Penicillin-Streptomycin (10378016, Thermo Fisher Scientific) in an incubator at 37°C and with an environment with 95% air and 5% ${\mathrm{CO}}_{2}$. The cells were imaged at room temperature one day after plating and within 30 minutes of removal from the incubator.^17^ The images were collected at 15 frames per second, and the dynamic contrasts were collected across 300 frames.

**Fig. 4:**
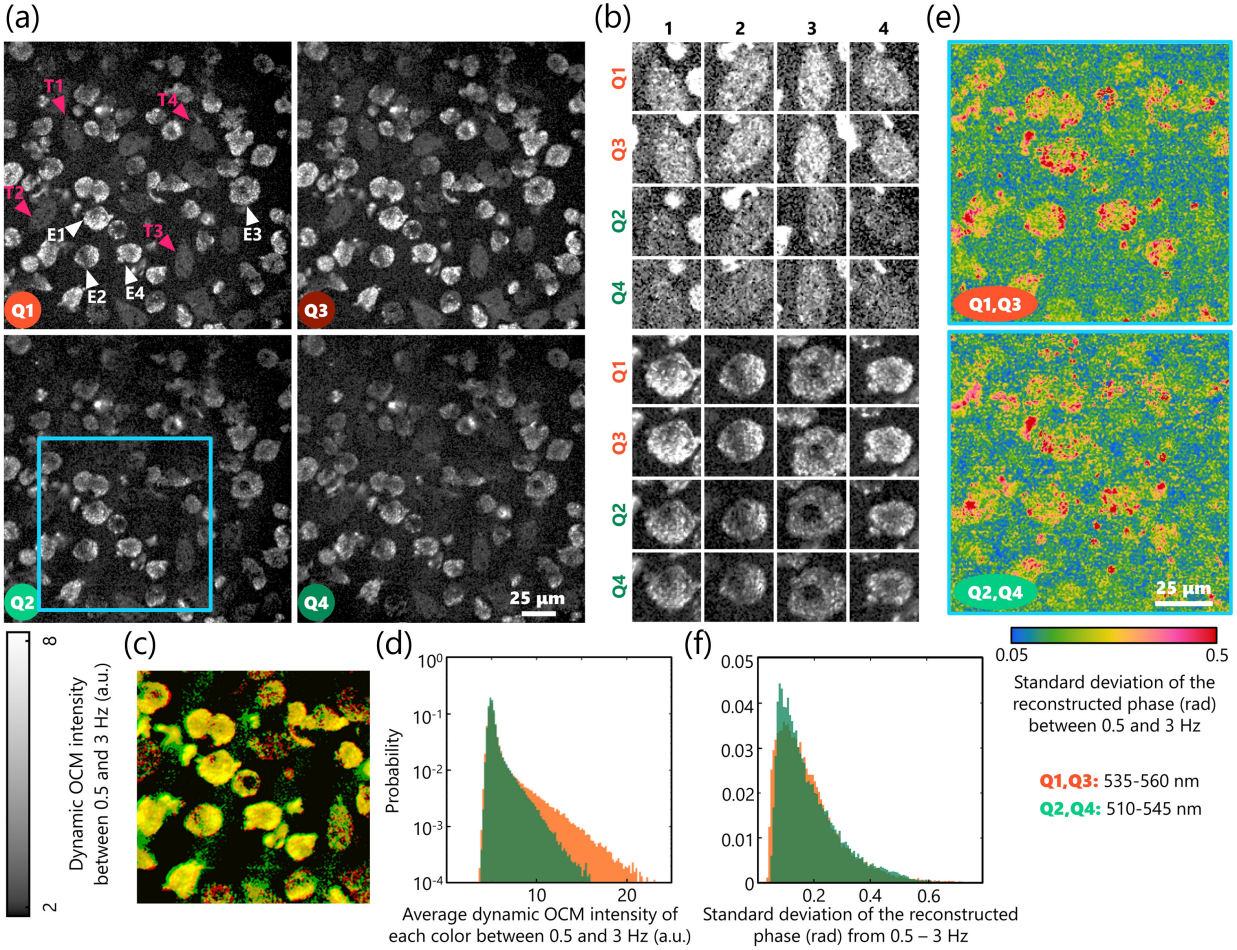
Imaging NE-4C cell dynamics with BiTe OCM. (a) Dynamic OCM intensity images of NE-4C cells in each quadrant between 0.5 and 3 Hz. The white and pink arrows highlight cells of two distinct populations: brighter round cells (in white, more active) and dimmer tear-drop-shaped cells (in pink, epithelial-like morphology). (b) Zoomed-in examples of the cells highlighted by the white and pink arrows in (a) in each quadrant, where the teardrop-shaped cells are shown in the top rows and the round cells are shown in bottom rows. The color scales of the two cell types were normalized for display, despite the cells in the top rows being five times dimmer than the ones in the bottom rows. (c) Pseudo-spectroscopic OCM image of the zoomed-in region of the NE-4C cells where the red channel corresponds to the average intensity in $Q_{1}$ and $Q_{3}$, and the green channel corresponds to the average intensity in $Q_{2}$ and $Q_{4}$ for the cells bound by the blue box in a. (d) Histogram of the OCM intensity of each color. (e) Reconstructed phase of each color based on the methods described in Fig. 2 shown for the region highlighted by the blue square in a. (f) Histogram of the reconstructed phase, showing negligible differences between the dynamics for the two colors of the cells, as expected. Scale bars: 25  μm.

Figure 4 highlights the results of imaging live NE-4C cells plated on a glass surface. First, neither dynamic OCM nor phase reconstruction shows any spatial interference effects from tilting the primary beam splitter. Second, imaging without the λ/8 waveplate improves the effective field-of-view from the previous iteration^24^ (1.25×) without sacrificing the quality of imaging subcellular dynamics. Two distinct populations of cells are apparent in Fig. 4(a) in each quadrant (indicated by the pink and white arrows). Previous studies have also linked the contrast in dynamic intensity from OCM to sub-cellular metabolic activity.^20^ One population appears less active and teardrop shaped, which is typical of epithelial cells. Since the cell line, NE-4C, has stem-like characteristics that can yield multiple phenotypes, there is another more active sub-population with round morphology apparent in these images [Fig. 4(b)]. The differences between the metabolism of different sub-populations of NE-4C cells during differentiation have been reported by Jády et al.^31^ validating our observations with BiTe OCM. Subcellular organelles and nuclei are apparent in these active sub-populations; overlaying the two colors over one another does not reveal any spectroscopic structural contrast [Fig. 4(c)], which was expected from these cell lines. Third, while the reconstructed phase from just two phase profiles is less accurate in comparison to using four orthogonal phase differences, the cellular morphology is distinguishable, nonetheless [Fig. 4(e)]. Particularly, the broader range of phases for the round subpopulation compared to the teardrop-shaped subpopulation confirms the flatter epithelial-like morphology of the latter. Finally, even in the phase profiles, there are no obvious spectroscopic differences, as expected, which was validated by their identical histograms in Fig. 4(f). These results highlight that BiTe OCM could simultaneously generate both dynamic intensity-based contrast and partially reconstructed phase profiles for living samples at two different colors.

### 3.3 Imaging Live Micro Animals in 3D with BiTe OCM

**Fig. 5:**
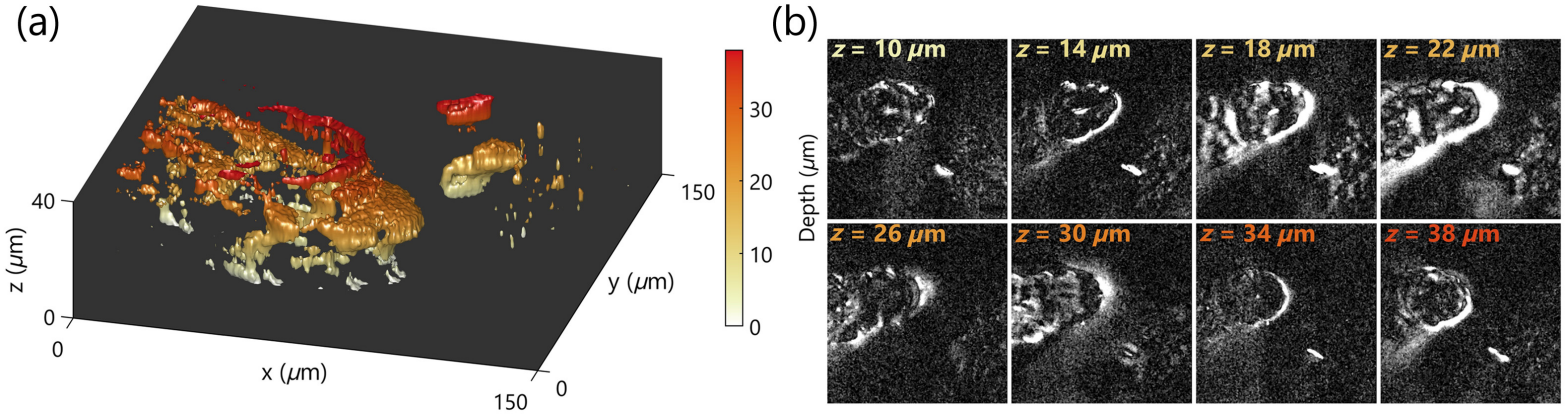
Imaging live tardigrade in 3D with BiTe OCM. (a) Reconstructed 3D object of a head section of a tardigrade, and (b) Selected planes from the reconstruction.

As a demonstration of the live and 3D imaging capabilities of BiTe OCM, we imaged freely moving tardigrades (*Hypsibius exemplaris*, Carolina Biological Supply) with our setup. Figure 5(a) shows a 3D rendered tardigrade head. Each slice is the dynamic OCM intensity of the high-frequency components (1.67 to $5.00\;s^{-1}$) computed from a time series of six frames acquired at 10 frames per second (total 4.8 s). The axial scanning was performed by moving the reference arm over this 30  μm range within this duration. Since the dynamic contrast was only calculated over a six-frame series, the image quality is considerably lower than that of the cells (which have the same order of refractive index mismatch with respect to these water bears). The depth sectioning offered by OCM enables 3D reconstruction of the water bear structures rapidly from individual slices [Fig. 5(b)], despite not restricting the motion of these animals. Being able to image the live organisms offers significantly higher dynamic contrast than the fringe contrast from OCM intensity alone. The axial sectioning offered by OCM enables rapid 3D imaging without moving the sample plane. This axial sectioning also allows tracking the micro animal across time rapidly while it is moving within the field of view (Video 1).

### 3.4 Imaging Tumor Dynamics with BiTe OCM

**Fig. 6:**
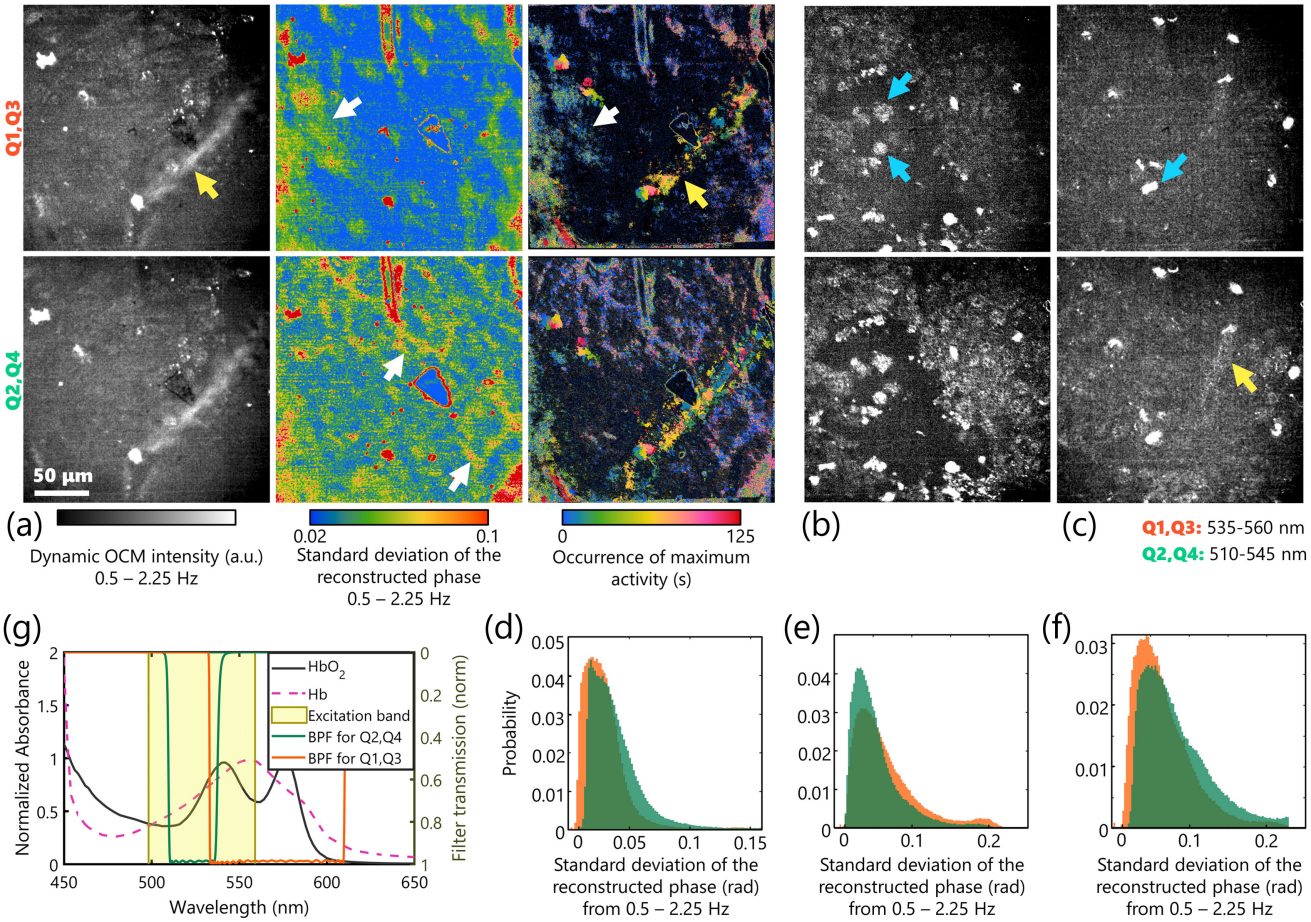
Imaging *ex vivo* tumor dynamics with BiTe OCM. (a) Dynamic OCM intensity between 0.5 and 2.25 Hz, standard deviation of the reconstructed phase between 0.5 and 2.25 Hz, and images of the occurrence of maximum activity estimated from abrupt changes to the reconstructed phase from adjacent frames of mammary tumor near the vasculature at each color. The yellow arrow indicates a large blood vessel apparent in the intensity images; the white arrow indicates smaller vessels apparent in the phase images. (b) and (c) Dynamic OCM intensity between 0.5 and 2.25 Hz of a mammary tumor away from (b) and near vasculature (c). (d)–(f) Histogram of the reconstructed phase for (a)–(c), respectively. (g) Normalized absorption spectra of oxygenated and deoxygenated hemoglobin [${\mathrm{HbO}}_{2}$ (black) and Hb (pink), respectively] overlaid on the excitation (yellow shaded area) and emission filters used in BiTe OCM (green and orange curves). The areas under the curve for ${\mathrm{HbO}}_{2}$ are 7.80 and 8.76, respectively, for the green and orange channels and for Hb are 8.61 and 8.76, respectively, for the green and orange channels. Therefore, the $O_{2}$ absorption ratio for the two channels were 1.11 and 1.00 for the green and orange channels, respectively. Scale bar: 250  μm.

An important advantage of OCM over other modalities is its ability to image deeper into scattering tissues. However, the previous iteration of the setup could not image through the superficial scattering layers due to the wavefront error caused by the variable waveplate, given the limited spatial coherence of the source. Having overcome that limitation in this study, BiTe OCM could image scattering samples like tumor tissues (Fig. 6). Approximately $9\times 10^{6}$ MAT B III rat mammary adenocarcinoma cells (CRL-1666, American Type Culture Collection) were grown in a media comprised of McCoy’s 5A (Modified) medium (16600082, Thermo Fisher Scientific) supplemented with 10% v/v fetal bovine serum (16140071, Thermo Fisher Scientific, MA) and 1% v/v Penicillin-Streptomycin (10378016, Thermo Fisher Scientific) and were injected subcutaneously into two healthy rats. After 7 or 8 days, when the tumor was $∼50\;{\mathrm{mm}}^{3}$, the rats were euthanized by ${\mathrm{CO}}_{2}$ asphyxiation. For each rat, immediately postmortem, the tumor was surgically resected and placed in an imaging dish with clear glass bottom containing ∼100  μL of freshly prepared phosphate-buffered saline and imaged with 5 min of extraction.

A few observations could be discerned from the images of the tumor shown in Fig. 6 that highlight the capabilities of BiTe OCM. First, as seen in the different fields of view in Figs. 6(a)–6(c), the dynamic contrast of BiTe OCM captures cellular (blue arrows), vascular (yellow arrows), and extracellular regions in the tumor distinctly. All images in Fig. 6 were captured ∼15  μm below the surface (see Fig. S1 in the Supplementary Material for a field of view captured 40  μm below the surface). The micron-scale transverse and axial resolutions could capture the individual cells of the tumor microenvironment and even capture some nuclear and subcellular structures [Fig. 6(b)]. Second, the reconstructed phase highlights vascular-rich regions in the sample, attributed to the cumulatively higher optical path length differences caused by the vasculature compared to the extracellular space. Third, structural differences in the two colors are apparent through visual observation of the dynamic OCM images. Specifically, due to the higher absorption of deoxygenated hemoglobin by the wavelength range (535 to 560 nm) of $Q_{1}$ and $Q_{3}$ [Fig. 6(g)],^32^ the structures appear comparatively dimmer for the cellular and extracellular region-rich locations in Figs. 6(b)–6(c). The reconstructed phase of the orange channel, which corresponds to $Q_{1}$ and $Q_{3}$ and has lower absorbance for oxygenated blood, has higher signals from the larger blood vessels that retain more oxygenated hemoglobin. In a vascular-rich region, where one expects a balance between the oxygenated and deoxygenated components for fresh samples,^33^ the disparity between the brightness in the two channels is lower. The reconstructed phase of the same region clearly highlights the two distinct kinds of blood vessels in the sample. The reconstructed phase of the green channel, which corresponds to $Q_{2}$ and $Q_{4}$ and has lower absorbance for deoxygenated hemoglobin, clearly highlights the microvasculature in the sample. The histograms of the phase profiles [Figs. 6(d)–6(f)] show that the presence of vasculature shifts causes comparatively higher signals in the green channel, whereas the cellular regions have nearly equal phase signals from both channels.

Video-rate imaging with BiTe OCM enables capturing and tracking the motion of individual objects, such as blood cells traveling through a vessel or cells moving within the extracellular space. These dynamics are apparent in Video 2, where the intensity and phase of each frame after filtering is overlayed on the average images for contextual information. Additionally, these dynamics were also visualized as the instance of maximum activity in the third column of Fig. 6(a). While the microvasculature and extracellular space demonstrate dynamics for the entire duration of imaging, the motion of larger objects appears as streaks of distinct colors that indicate spatially and temporally localized instances of activity. The results in this section demonstrate that the different contrasts of BiTe OCM, coupled with video-rate axially sectioned imaging, could capture various cellular and vascular dynamics of the scattering tumor tissue.

### 3.5 Imaging Fall Foliage with BiTe OCM

Apart from its suitability for hemodynamic spectroscopy, the two colors of BiTe OCM are ripe for imaging the various dynamics of fall foliage. Imaging the leaves with BiTe OCM can help relate the microstructural heterogeneity of the plant cells to the macroscale colors. Additionally, BiTe OCM can generate axially sectioned profiles of the cells to observe the different depths within a leaf distinctly. Leaves of different colors were collected from sugar maple (*Acer saccharum*), beech (*Fagus grandifolia*), and mulberry (*Morus rubra*) trees outside the Beckman Institute for Advanced Science and Technology in Urbana, IL, in early November, during the fall season. A small square of each leaf was cut and placed in an imaging dish with approximately 100  μL of distilled water. A metal washer was placed on top of the leaf to ensure contact with the glass surface at the bottom of the imaging dish. All leaves were imaged within 15 min of removal from the trees.

**Fig. 7:**
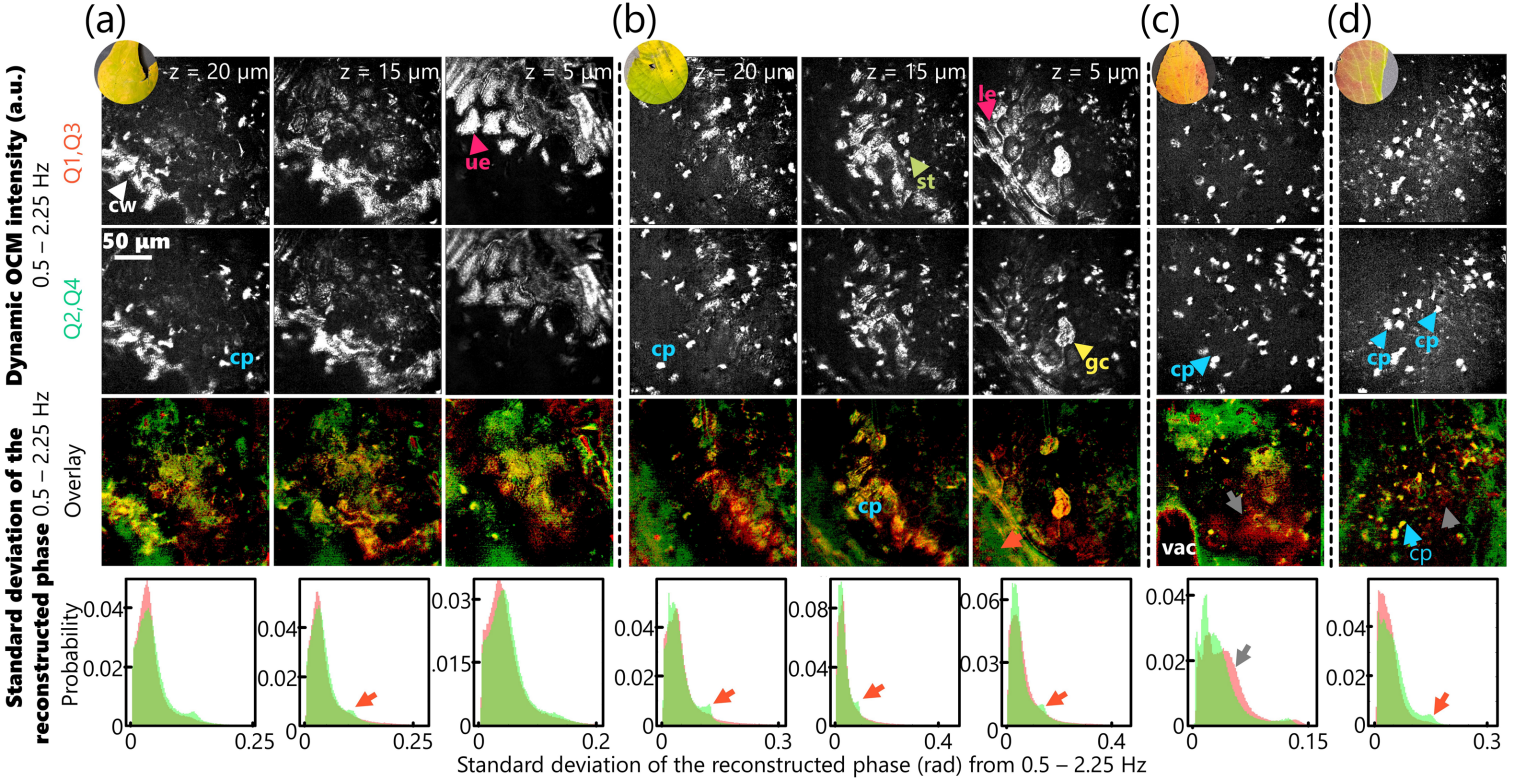
Imaging fall foliage with BiTe OCM. Dynamic OCM images of four leaves of varying colors and overlaid images of the standard deviation of the reconstructed phase for each color, $Q_{1}$ and $Q_{3}$ in red, and $Q_{2}$ and $Q_{4}$ in green. The histograms correspond to the distributions of the respective standard deviation of the reconstructed phase for each color. The orange and grey arrows indicate regions with higher signals in the green and red channels, respectively. The pink arrows indicate the epiderma (UE: upper epidermis and LE: lower epidermis); the green arrows indicate stroma (st), the yellow arrows indicate guard cells (gc), the blue arrows indicate chloroplasts (cp), cell walls (cw), and vacuoles (vac) are marked in white. The round insets near the top correspond to macroscopic photographs of each leaf used to image. (a) and (b) highlight the 3D images obtained at three different depths using BiTe OCM. Scale bar: 50  μm.

Figure 7 highlights the images captured from these leaves. First, since the parenchymal cells of plants are substantially larger than mammalian cells, around 100 to 200  μm, the field-of-view of the setup is approximately the same as the size of a one or two cells.^34^ Second, the microscale structure of each leaf shown in Fig. 7 has drastically different features, even at the same depth. The epidermis appears honeycomb-like in the leaf in Fig. 7(b), the epidermis of the leaf in Fig. 7(a) is less organized.^35^ Moreover, since the leaf in Fig. 7(b) was imaged from the under side, the guard cells appear bright in the intensity images because they are dynamically active and have higher refractive index mismatch due to water resorption.^36^ While most subcellular organelles of the plant have minimal refractive index mismatch or dynamic contrasts, the chloroplasts, which have a higher starch and water content, appear as bright objects within the cytoplasm in BiTe OCM images. Video 3 shows a time-lapse of the dynamic OCM intensity of the two channels for leaves imaged over 24 s.

Third, the spectroscopic contrast is not obvious in the intensity images; however, the reconstructed phase highlights these differences. Since chlorophylls, which are concentrated in chloroplasts, show minimal absorption throughout the imaging spectral width, they appear as “yellow” structures with nearly equal absorption and scattering in both channels. Since vacuoles are not dynamic or highly scattering, they have minimal contrast in dynamic OCM. However, the vacuole inside the cell in the leaf shown in Fig. 7(c) appears distinct with the phase contrast. Unlike chlorophyll, carotenes, which are also present in chloroplasts, absorb green light much more than yellow or orange colors.^37^ Therefore, carotene-rich regions are expected to appear reddish (due to lower absorption) in these images. The leaf in Fig. 7(c), the least “green” in the photograph, also has maximal red regions in the phase images, validated by the peak indicated by the grey arrows in the histograms. Alternatively, the other leaves that appear greener in the photographs [Figs. 7(a), 7(b), and 7(d)] have higher signals in the green regions, validated by the peak indicated by the orange arrows in the histograms. These results further highlight the ability of BiTe OCM to obtain pseudo-spectroscopic 3D images of dynamic living samples in the visible spectrum.

## 4 Discussion

BiTe OCM overcomes several disadvantages of previous techniques for FF-OCM. Compared to the serial acquisition of typical time-domain FF-OCM setups, BiTe OCM acquires all raw data with a single-shot acquisition. While off-axis OCM setups offer single-shot acquisition, the necessary high spatial coherence induces speckle noise in the resulting images.^17^^,^^18^ Alternatively, hyperspectral cameras^13^ and light-field tomography^38^ have been used for 3D snapshot FF-OCM. Our previous implementation^24^ of four-phase-shifted OCM utilized a variable waveplate to create four unique phase shifts. Each used a spatially incoherent source to generate OCM images of samples with a single scattering surface. However, snapshot FF-OCM techniques could not image scattering samples such as biological tissues since the photon efficiency of the hyperspectral cameras was low. Moreover, these techniques also have several artifacts from the hyperspectral or light-field detection schemes. In the case of four-phase-shifted OCM, the wavefront errors caused by the variable waveplate reduced the fringe contrast for the low spatially coherent source in scattering samples. Dynamic OCM could overcome these issues and generate high-resolution images of living biological samples by sacrificing complete optical field reconstruction. However, BiTe OCM overcomes the disadvantages of all these techniques by capturing both the intensity and phase profiles with a single-shot acquisition, without any artifacts, without speckle noise by using a spatially low coherence source, with the ability to image scattering 3D samples.

One limitation of BiTe OCM is that the field of view is limited to one quarter of the sensor size in order to accommodate four quadrants in the image plane. In this study, the field-of-view was restricted to a maximum of $200\times 200\;\mu m^{2}$. Apertures were placed on conjugate planes on the source side and before the visible-coating PBS to restrict overlap between the different quadrants. In this implementation of BiTe OCM and in the previous setup,^24^ the beams were deliberately directed into a single camera, where this field-of-view was sufficient for the demonstrations. The beams could be directed into four individual cameras sharing a common acquisition external trigger, utilizing the field-of-view of each completely. This would have the added advantage of equalizing the sample and reference powers at each wavelength sub-band and polarization. This disparity in the optical powers arises from using the spectrally mismatched AR coatings. From the manufacturer’s data provided for normal incidence, the transmittance at 510–545 nm was higher for both polarization states (25% to 50% for P-polarized and 15% to 20% for S-polarized light) than 535 to 560 nm (20% to 30% for P-polarized and 13% to 17% for S-polarized). If this trend holds for non-normal incidence, a drastic power difference is expected for each quadrant. This was minimized in two ways. First, the choice of the LED used in this study had peak intensity at 565 nm. Therefore, the higher transmittance at the lower wavelength sub-band was negated by the lower power within this wavelength sub-band. Second, the filters were placed in such a way that the wavelength sub-band was matched with the polarization state that encountered more losses. This ensured that when imaging a mirror in either the sample or reference arms, the intensity on the camera was <20% difference between two different quadrants, on average.

BiTe OCM, to our knowledge, is the first utilization of AR coatings to create phase shifts in the beams. The phase shifts are not mutually orthogonal; the problem of complete reconstruction of the optical phase is, therefore, ill-posed.^39^ In BiTe OCM, there is an underlying assumption that the intensity of the scattered field from the sample is minimal and that the cross-correlation between the two optical fields could be normalized with respect to the background. However, this assumption is not entirely accurate. Therefore, the reconstructed pseudo phase does contain some elements of the intensity of the scattered field, where the phase profile of highly scattering features appears more prominent. This dependency is apparent in the reconstructed phase of the NE-4C cells, where the phase profile appears specular rather than the otherwise smoother profile expected. In contrast, in scattering samples such as tumors or leaves, the reconstructed pseudo phase exclusively highlights features not apparent in the dynamic intensity. For instance, the vasculature, cell wall, and vacuoles are only visible in the reconstructed phase profile. Additionally, the aspects of intensity changes encoded in the reconstructed phase accentuate the spectroscopic contrasts, which are largely driven by differences in the absorbances at the two spectral bands. However, the phase was also quantitatively retrieved in Fig. 3 providing a complete solution despite only having reliable performance for uniformly scattering samples. In brightly scattering samples, when $E^{2}(x,y)\neq 0$, Eq. (2) does not have a deterministic solution of the form in Eq. (3). However, it can be solved numerically by considering Eq. (2) as quadratic and nonlinear equations of the intensity and phase.

Our implementation of BiTe OCM in this paper is but one possible version of the setup, which used off-the-shelf optical elements and a readily available low-cost LED source. As a proof-of-concept, we utilized the phase-shifting properties of existing AR coatings. However, with a more careful design of the AR coatings and by tuning the tilt angle of the primary beam splitter, there is a configuration of BiTe OCM where the phase differences could be mutually orthogonal. In this case, a single quantitative reconstruction of the optical phase is possible for samples with minimal spectroscopic contrast. Another aspect of BiTe OCM that is application-specific is the light sources and filters used. The combination presented in this paper was chosen specifically for the two applications in Figs. 6 and 7. BiTe OCM only requires that the light source have low coherence, both spatially and spectrally. Other sources or wavelength bands could be more appropriate for other applications, such as using red or NIR regions to image deeper into tissues. Unlike other OCT modalities, such as swept source or spectral-domain sources that need optically amplified radiation and, therefore, need more drastic redesign to adapt the setup to a different spectral band, LED sources across the UV-Vis-NIR spectrum are cheaper and prevalent. The LED used in this setup also had a very low spatial coherence factor, which was calculated as the ratio of the standard deviation to the mean intensity across a field-of-view of a clean mirror surface to be −23  dB. This restricted the maximum fringe contrast from a clean glass–air interface to 17.4% to 20.3% (for each quadrant) of the reference signal. This restricted the imaging depths in scattering samples to a few dozen microns. The mammary tumor imaged 40  μm below the surface, shown in Fig. S1 in the Supplementary Material, has a signal-to-noise ratio of just 10 dB, compared with 30 dB at the surface for the OCM intensity (although the dynamic contrast was much higher). The imaging speed was also limited by the fluence of the LED used in this study for the scattering profiles of these samples. The LED also had a relative standard deviation of −30.5  dB, measured from a time-series of 64 frames from the reference mirror surface, which led to incorrect background subtraction and causes an apparent “blinking” effect in the videos. This could be mitigated by scaling the background based on an independent monitoring of the LED output variations. At the cost of losing the versatility of using an LED, using other incoherent sources that are fiber based with higher radiance, such as fiber-based amplified spontaneous emission light,^40^ could also improve the photon budget and have higher spatial coherence, thereby, enabling faster and deeper imaging, respectively. The frequency bands chosen for the dynamic OCM contrast in this study were also chosen based on the balancing the maximum frame rate based on the source fluence and the high-frequency noise of the source with maximizing the information from the sample. A better source would enable dynamic contrasts in different frequency bands to differentiate multiple components within the sample.^21^

The applications of pseudo-spectroscopy presented in this paper are only a subset of the possible applications. Imaging the blood oxygenation with OCT is well-known,^28^^,^^29^ albeit for other OCT/ OCM configurations in different contexts. The extraction of quantitative oxygenation measurements from tissues requires adapting BiTe OCM for *in vivo* mammalian imaging and overcoming the restrictions of imaging depths, which will be explored in future studies. However, the utility of dynamic OCM for plant imaging is relatively underexplored. The utility of OCT in post-harvest quality evaluation has previously been proposed^41^; several high-throughput systems have been explored in this space for structure-based evaluations of fruits,^42^^,^^43^ vegetables,^44^^,^^45^ and grains,^46^ and grains.^46^ While OCT has been used to study the 3D microstructure plants for over two decades,^47^^–^^49^ dynamic imaging of plants has remained underexplored, except few recent demonstrations.^50^^,^^51^ The growing need for imaging plant dynamics^52^ for high-throughput commercial crop evaluation could be filled by dynamic OCM; the pseudo-spectroscopy in BiTe OCM provides a useful complementary contrast. Additionally, the four quadrants in BiTe OCM were also detected for two different polarization states. An alternative extension of functional OCM could swap spectroscopic contrast for polarization contrast depending on the application. The fundamental framework of BiTe OCM can be readily adapted to various applications without the need for any specialized optical elements.

## 5 Appendix: Video Information

Video 1. Imaging a moving tardigrade with BiTe OCM, showing the dynamic OCM intensity from the high-frequency components from six frames in a time series acquired at 10 frames per second over 90 s. Scale bar: 20  μm (MP4, 2.85 MB [URL: https://doi.org/10.1117/1.JBO.29.S2.S22704.s1]).

Video 2. Imaging *ex vivo* tumor dynamics with BiTe OCM. (Left) Real part of the OCM intensity and (Right) reconstructed OCM phase between 0.36 and 3.74 Hz for each color, where each frame, visualized as a heat map, is overlayed on the average dynamic OCM image of all frames (MP4, 16.0 MB [URL: https://doi.org/10.1117/1.JBO.29.S2.S22704.s2]).

Video 3. Imaging the dynamics within leaves with BiTe OCM. Each frame shows the dynamic intensity of the leaf over 40 frames in each (red and green) channel (MP4, 19.5 MB [URL: https://doi.org/10.1117/1.JBO.29.S2.S22704.s3]).

## Supplementary Material









## Data Availability

The raw data that support the findings of this study are available from the corresponding author upon request. The codes used to process the images are available at 10.6084/m9.figshare.25413955. No new materials were generated in this study.
