# Supplementary material for: Bichromatic tetraphasic full-field optical coherence microscopy
Source: J Biomed Opt. 2024 Mar 25;29(Suppl 2):S22704. doi: 10.1117/1.JBO.29.S2.S22704 (PMC10996847; doi:10.1117/1.JBO.29.S2.S22704)
Supplement: Supplementary file 1 [file JBO_029_S22704_SD001.pdf]

Bichromatic Tetraphasic Full-Field Optical Coherence Microscopy: supplemental document

Note S1:

Derivation for the reconstruction of phase for each color

Description of variables:

$I_{Q_x}^{U}\left(x,y\right)$	Detected OCM image after unwarping in quadrant Q_x
$B_{Q_x}^{U}\left(x,y\right)$	Background image from the reference along x and y in quadrant Q_x
$\phi\left(x,y\right)$	Sample phase along x and y
$S_{Q_x}^{U}\left(x,y\right)$	Scattered intensity from the sample along x and y in quadrant Q_x
$\delta_{Q_x}\left(y\right)$	Phase difference between sample and reference $y$in quadrant Q_x
S_{Q_x}	Average $I_{Q_x}^{N}$ along x, y and t in quadrant Q_x

In BiTe OCM, the normalized intensity in each quadrant was derived as:

$$I_{Q_x}^{N}\left(x,y\right)=\frac{I_{Q_x}^{U}\left(x,y\right)-B_{Q_x}^{U}\left(x,y\right)}{\sqrt{B_{Q_x}^{U}\left(x,y\right)}}. \tag{S1}$$

Since the detected OCM intensity, $I_{Q_x}^{U}\left(x,y\right)$ is the interference between the sample and reference, Eq. S1 can be written as

$$I_{Q_x}^{N}\left(x,y\right)=\frac{\left(\begin{array}{c}S_{Q_x}^{U}\left(x,y\right)+B_{Q_x}^{U}\left(x,y\right)+\\ 2\sqrt{S_{Q_x}^{U}\left(x,y\right)B_{Q_x}^{U}\left(x,y\right)}\cos\left(\phi\left(x,y\right)+\delta_{Q_x}\left(y\right)\right)\end{array}\right)-B_{Q_x}^{U}\left(x,y\right)}{\sqrt{B_{Q_x}^{U}\left(x,y\right)}}. \tag{S2}$$

Assuming comparatively negligible scattering from the sample and that the intensity contrast from the sample scattering is minimal along x and y, Eq. S2 can be reduced to

$$\begin{aligned}I_{Q_x}^{N}\left(x,y\right)&\approx\frac{\left(B_{Q_x}^{U}\left(x,y\right)+2\sqrt{S_{Q_x}^{U}\left(x,y\right)B_{Q_x}^{U}\left(x,y\right)}\cos\left(\phi\left(x,y\right)+\delta_{Q_2}\left(y\right)\right)\right)-B_{Q_x}^{U}\left(x,y\right)}{\sqrt{B_{Q_x}^{U}\left(x,y\right)}}\\&=2\sqrt{S_{Q_x}^{U}\left(x,y\right)}\cos\left(\phi\left(x,y\right)+\delta_{Q_x}\left(y\right)\right)\\&\approx S_{Q_x}\cos\left(\phi\left(x,y\right)+\delta_{Q_x}\left(y\right)\right)\end{aligned} \tag{S3}$$

Since Q_2 and Q_4 are from the same color, multiplying the two normalized intensities in Eq. S3 can be used to derive the expression for the retrieved sample phase at this color.

$$I_{Q_2}^{N}(x,y)I_{Q_4}^{N}(x,y)=S_{Q_2}S_{Q_4}\cos\left(\phi(x,y)+\delta_{Q_2}(y)\right)\cos\left(\phi(x,y)+\delta_{Q_4}(y)\right)=\frac{S_{Q_2}S_{Q_4}}{2}\begin{pmatrix}\cos\left(2\phi(x,y)+\delta_{Q_2}(y)+\delta_{Q_4}(y)\right)\\+\cos\left(\theta_{Q_2-Q_4}(y)\right)\end{pmatrix} \quad \text{(S4)}$$

$$\frac{2I_{Q_2}^{N}(x,y)I_{Q_4}^{N}(x,y)}{S_{Q_2}S_{Q_4}}-\cos\left(\theta_{Q_2-Q_4}(y)\right)=\cos\left(2\phi(x,y)+\delta_{Q_2}(y)+\delta_{Q_4}(y)\right)=\cos\left(\phi_{510-545\text{ nm}}(x,y)\right) \quad \text{(S5)}$$

$$\cos\left(\phi_{510-545\text{ nm}}(x,y)\right)=\frac{2I_{Q_2}^{N}(x,y)I_{Q_4}^{N}(x,y)}{S_{Q_2}S_{Q_4}}-\cos\left(\theta_{Q_2-Q_4}(y)\right)$$
$$\phi_{510-545\text{ nm}}(x,y)=\cos^{-1}\left(\frac{2I_{Q_2}^{N}(x,y)I_{Q_4}^{N}(x,y)}{S_{Q_2}S_{Q_4}}-\cos\left(\theta_{Q_2-Q_4}(y)\right)\right) \quad \text{(S6)}$$

The same logic can be applied to quadrants 1 and 3 to yield the expressions in Eq. 2.

Figure S1

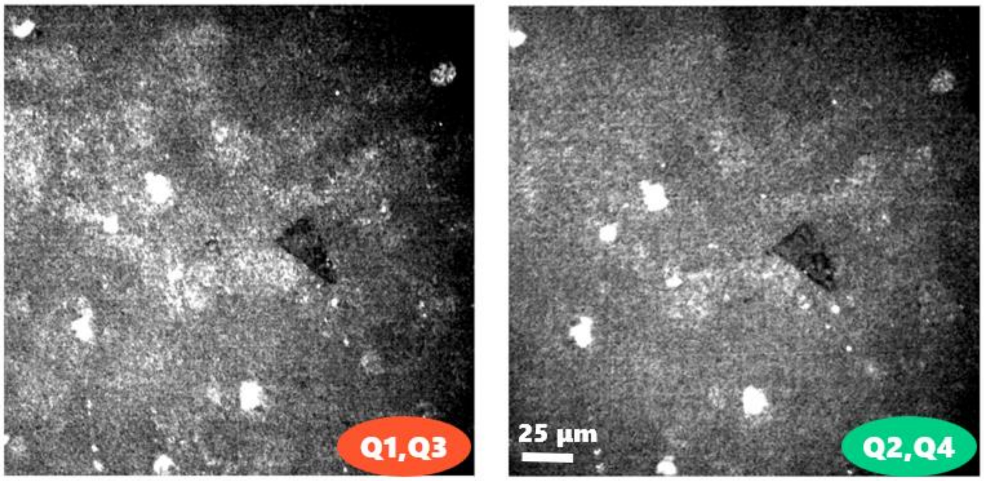

Figure S1. Dynamic OCM intensity of rat mammary tumor imaged 40 µm below the surface; related to Figure 3.
